# Patient‐reported symptom burden as a prognostic factor in treatment with first‐line cetuximab plus chemotherapy for unresectable metastatic colorectal cancer: Results of Phase II QUACK trial

**DOI:** 10.1002/cam4.2826

**Published:** 2020-01-21

**Authors:** Akira Ooki, Satoshi Morita, Shigeyoshi Iwamoto, Hiroki Hara, Hiroaki Tanioka, Hironaga Satake, Masato Kataoka, Masahito Kotaka, Yoshinori Kagawa, Masato Nakamura, Tatsushi Shingai, Masashi Ishikawa, Yasuhiro Miyake, Takeshi Suto, Yojiro Hashiguchi, Taichi Yabuno, Junichi Sakamoto, Akihito Tsuji, Masahiko Ando, Kensei Yamaguchi

**Affiliations:** ^1^ Department of Gastroenterological Chemotherapy Cancer Institute Hospital of Japanese Foundation for Cancer Research Tokyo Japan; ^2^ Department of Biomedical Statistics and Bioinformatics Kyoto University Kyoto Japan; ^3^ Cancer Center Aichi Medical University Nagakute Japan; ^4^ Department of Gastroenterology Saitama Cancer Center Saitama Japan; ^5^ Department of Clinical Oncology Kawasaki Medical School Kurashiki Japan; ^6^ Cancer Treatment Center Kansai Medical University Hospital Osaka Japan; ^7^ Department of Surgery National Hospital Organization Nagoya Medical Center Nagoya Japan; ^8^ Gastrointestinal Cancer Center Sano Hospital Kobe Japan; ^9^ Department of Surgery Kansai Rosai Hospital Amagasaki Japan; ^10^ Comprehensive Cancer Center Aizawa Hospital Matsumoto Japan; ^11^ Department of Surgery Osaka Saiseikai Senri Hospital Suita Japan; ^12^ Department of Surgery Shikoku Central Hospital Shikokuchuo Japan; ^13^ Department of Surgery Osaka Minato Central Hospital Osaka Japan; ^14^ Department of Surgery Yamagata Prefectural Central Hospital Yamagata Japan; ^15^ Department of Surgery Teikyo University School of Medicine Tokyo Japan; ^16^ Department of Surgery Yokohama Municipal Citizen's Hospital Yokohama Japan; ^17^ Tokai Central Hospital Kakamigahara Japan; ^18^ Department of Medical Oncology Kagawa University Kita Japan; ^19^ Department of Advanced Medicine Nagoya University Hospital Nagoya Japan

**Keywords:** cetuximab, chemotherapy, colorectal cancer, quality of life, symptom

## Abstract

**Background:**

It remains unclear whether patients’ self‐perceptions of symptoms at baseline clinically impact the prognostic relevance, treatment efficacy, or toxicity profiles in metastatic colorectal cancer (mCRC) patients treated with the first‐line cetuximab and standard chemotherapy.

**Methods:**

The data were collected from a prospective trial that assessed the relationships between quality of life (QOL), treatment efficacy, and adverse events (AEs).

**Results:**

The analysis of 137 mCRC patients revealed a significant association between the presence of baseline tumor‐related symptoms and a lower overall survival (OS) compared to the absence of symptoms (HR, 2.49; 95% CI, 1.37‐4.62; *P* = .003). The asymptomatic responders had favorable outcomes compared to the symptomatic nonresponders (2‐year OS rates: 83.6% and 35.9%, respectively), while the symptomatic responders had similar outcomes to the asymptomatic nonresponders. The median postprogression survival differed significantly: 10.2 months for the symptomatic patients and 15.9 months for the asymptomatic patients (HR, 2.29; 95% CI, 1.25‐4.29, *P* = .008). The objective response rates and patient toxicity profiles were similar irrespective of the severity of baseline symptoms.

**Conclusion:**

Baseline symptoms were associated with worse OS but not with impaired treatment efficacy or more frequent AEs in mCRC patients treated with cetuximab in addition to chemotherapy.

## Graphical abstract

This study provides that the baseline patient‐reported symptoms are associated with worse OS, but not with impaired treatment efficacy or deteriorated AEs, for patients treated with cetuximab plus chemotherapy.

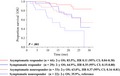

## 1 INTRODUCTION

As colorectal cancer (CRC) is the third leading cause of cancer‐related deaths in the USA, it remains a major clinical challenge.^1^ Approximately 25% of patients with CRC is diagnosed as metastatic disease.^2^ Chemotherapy commonly comprises a doublet of cytotoxic agents (fluoropyrimidine plus irinotecan or oxaliplatin) and is the cornerstone treatment for metastatic CRC (mCRC) patients.^3^ Anti‐epidermal growth factor receptor antibodies (anti‐EGFR ab: cetuximab and panitumumab) have demonstrated significant survival advantages,^4^ and combining them with chemotherapy is one of the most promising primary treatment regimens for left‐sided mCRC patients with RAS wild type.^5^, ^6^, ^7^ Despite advances in treatments for unresectable mCRC, the main goal of treatment is generally palliative rather than curative.^8^ Accordingly, in addition to the prevention of cancer progression and the prolongation of patient survival, consideration should be given to both the improvement of tumor‐related symptoms and the maintenance of health‐related quality of life (HRQOL) in treatment planning.

The conceptual framework of HRQOL includes the three dimensions of social, mental, and physical well‐being, all of which are affected by the burden of a patient's symptoms.^9^, ^10^, ^11^ Consequently, HRQOL is a subject of major concern for patients with mCRC due to frequently occurring symptoms, such as constipation, pain, fatigue, and appetite loss.^12^ Patient‐reported outcomes (PROs) are considered to be any reports on the status of a patient's health condition that come directly from the patient without any interpretation of the patient's response by a clinician nor anyone else.^13^ Since substantial variability exists between physicians’ and patients’ assessments of symptoms, especially for more subjective items,^14^, ^15^ PROs are becoming increasingly crucial when determining the subjective aspects of a patient's HRQOL. In addition to providing more accurate assessments of symptom severity, PROs have recently emerged not only as a key prognostic factor across different cancer types including CRC,^16^, ^17^, ^18^ but also as a predictor of chemotherapy benefit and adverse events (AEs) in women with advanced breast cancer.^19^ Therefore, PROs will play an important role in routine clinical practice when assessing the balance between the expected benefits and possible risks of treatment. However, a lack of such valuable information remains a serious problem in the treatment of mCRC.

Recently, we demonstrated in the prospective Phase II QUACK study^20^, ^21^ that severe early skin toxicities are associated with favorable overall survival (OS) rates but without impairing HRQOL for patients treated with cetuximab and chemotherapy, as evaluated by the European Organization for Research and Treatment of Cancer (EORTC) Quality of Life Questionnaire C30 (QLQ‐C30), which is a valid and reliable HRQOL instrument in advanced cancer settings.^22^, ^23^, ^24^ In addition, patients who were symptomatic at baseline and who responded to treatment experienced improved HRQOL. The purpose of this study is to assess the association of patient‐reported baseline symptoms with prognosis, therapeutic efficacy, and toxicity using data from the QUACK study. The HRQOL at baseline for 137 of 140 patients enrolled from 49 institutions in Japan between July 2013 and April 2015 was used in this study. Our findings may constitute relevant additional information that will facilitate informed decision‐making and clinical management.

## 2 PATIENTS AND METHODS

### 2.1 Study design and treatment

The QUACK study is a prospective, multicenter, Phase II trial to investigate the associations between QOL, treatment efficacy, and toxicities in the primary treatment of mCRC with a combination of cetuximab and standard chemotherapy (FOLFOX or FOLFIRI). Detailed information with respect to the study's design has been previously described.^20^ One hundred and forty‐nine mCRC patients were enrolled from 49 institutions between July 2013 and April 2015 (Table S1). Nine patients were terminated from the study before the first administration of the study treatment, and 140 patients received the first‐line cetuximab plus chemotherapy.^21^ The FOLFIRI plus cetuximab regimen consisted of an initial infusion of 400 mg/m^2^ of cetuximab, followed by a weekly infusion of 250 mg/m^2^, with the concurrent administration of 200 mg/m^2^ of l‐leucovorin and 150 mg/m^2^ irinotecan; this was followed by an intravenous bolus of 400 mg/m^2^ of 5‐fluorouracil, with a continuous infusion of 2400 mg/m^2^ for 46 hours every 14 days. The mFOLFOX6 plus cetuximab regimen was the same as the regimen of FOLFIRI plus cetuximab, except that irinotecan was replaced with 85 mg/m^2^ of oxaliplatin. This study was conducted in accordance with the Declaration of Helsinki and the Ethics Guidelines for Clinical Research by the Ministry of Health, Labor, and Welfare in Japan. All patients provided written informed consent before registration. The study protocol was approved by the institutional review board or ethics committee of each participating institution and was registered with the University Hospital Medical Information Network (UMIN) Clinical Trial Registry (UMIN000010985) on July 19, 2013.

### 2.2 Safety and treatment efficacy

AEs were monitored to assess safety using laboratory and physical examinations, and the National Cancer Institute's Common Terminology Criteria (NCI‐CTCAE) version 4.0 was used to grade AE severity. The survey sheets, which included information regarding compliance with treatment, treatment efficacy, and safety, were collected at registration and after 4, 8, 16, and 24 weeks.

Radiological methods (computed tomography and/or magnetic resonance imaging) were performed to assess treatment efficacy prechemotherapy (baseline) as well as every eight weeks during the treatment period. The Response Evaluation Criteria in Solid Tumors (RECIST) version 1.1 was used to evaluate treatment response by the investigator at each institution. A responder is defined as a patient with a complete response (CR) or a partial response (PR) according to the RECIST following treatment with cetuximab plus chemotherapy. Progression‐free survival (PFS) is defined the time from registration to the time of progression after the initiation of first‐line treatment or the time of death from any cause. OS is defined as the time from registration until death. Postprogression survival (PPS) refers to the time from tumor progression after the initiation of first‐line treatment until the time of death, obtained by subtracting PFS from OS.^25^

### 2.3 HRQOL and symptom assessments

Previous trials, including QOL analyses, were not assessed as a primary endpoint and resulted in less frequent assessments of QOL. For example, in the CRYSTAL study, adding cetuximab to first‐line chemotherapy in patients with KRAS wild‐type mCRC, QOL was assessed every 8 weeks and at final tumor assessment.^26^ However, skin reactions are one of the most common cetuximab‐related AEs,^27^ and typical time courses of onset depend on different dermatologic side‐effects; acneiform skin rash and pruritus generally develop within the first three weeks of therapy, and xerotic skin and nail changes occur in 4‐8 weeks and after 4‐8 weeks, respectively.^4^, ^28^ Since this study was specifically designed to investigate associations between QOL and AEs, including skin toxicity reactions as a primary endpoint, frequent measurement of QOL during treatment was performed to assess the early and late impact of AEs on QOL; QOL assessments were performed at baseline and after 2, 4, 8, 16, and 24 weeks, and a window of two weeks around each follow‐up QOL assessment was accepted. If the prescribed treatment was not completed, the last assessment was done when the termination of the study was being determined or during the next scheduled appointment. HRQOL and symptom burden were assessed using the EORTC QLQ‐C30 version 3.0, which is a self‐administered, cancer‐specific, multidimensional questionnaire.^22^, ^23^ The EORTC QLQ‐C30 questionnaire is composed of both multi and single‐item scales, including a global health status (GHS)/QOL scale, five functional scales (cognitive, role, emotional, social, and physical), financial difficulties items, and eight symptom scales (nausea/vomiting, fatigue, constipation, dyspnea, pain, appetite loss, diarrhea, and insomnia).^22^ The observed raw scores were standardized by a linear transformation, so that scores range from 0 to 100; a higher score represents better levels of GHS/QOL and functioning, or a worse level of symptoms.^29^ A change in scale of at least 10 points is considered to be clinically relevant.^4^, ^30^ The EORTC QLQ‐C30 symptom items have four response categories (not at all, a little, quite a bit, very much). OS was significantly longer in patients who were asymptomatic at baseline compared with those who were symptomatic at baseline in the Phase III CRYSTAL study adding cetuximab to first‐line chemotherapy, in which patients were defined as symptomatic if they answered “quite a bit” or “very much” to at least one of the symptom questions of EORTC QLQ‐C30 at baseline and asymptomatic if they answered “not at all” or “a little” to all of the symptoms.^31^ To validate the association between symptoms at baseline and OS in an independent cohort, the same definition was used in this study.

### 2.4 Statistical analysis

Patients who withdrew consent before intervention were excluded from all analyses. QOL was analyzed for patients with both a baseline QOL assessment and at least one postbaseline QOL assessment. To estimate the distribution of the prognostic outcomes, the Kaplan‐Meier method was performed, and the log‐rank test was used to compare the distribution of survival times. The Cox proportional hazard model was used to analyze the association between the baseline symptoms and the time‐to‐event endpoints, and the adjusted hazard ratios (HR) and the 95% confidence intervals (CI) were calculated. In addition, multivariable Cox proportional hazard models were used to estimate the prognostic values of baseline symptoms on OS, adjusted for other factors that were statistically significant for the univariate analysis of OS. The continuous variable data were expressed as a mean ± the standard error of the mean (SEM). The continuous variables between symptomatic and nonsymptomatic patients were compared using a two‐tailed Student's t test, and Fisher's exact test was used for categorical variables. All statistical analyses were conducted using the JMP 12 software package (SAS Institute).

## 3 RESULTS

### 3.1 Clinicopathological characteristics of symptomatic patients at baseline

The HRQOL at baseline for 137 of 140 patients was assessed using the EORTC QLQ‐C30. High questionnaire compliance rates were maintained throughout the study period (ie, 97.9% at baseline, 96.2% at eight weeks, and 81.1% at 24 weeks for the QLQ‐C30).^21^ With regard to symptoms at baseline, 55 out of 137 patients (40.1%) reported at least “quite a bit” when assessing the severity of tumor‐related symptoms in the eight symptom items (ie, nausea/vomiting, fatigue, constipation, dyspnea, pain, appetite loss, diarrhea, and insomnia) on the EORTC QLQ‐C30 (Table S2). Compared with the asymptomatic patients, symptomatic patients had significantly lower GHS/QOL scores (median scores: 68.1 and 49.1, *P* < .0001), a worsened Eastern Cooperative Oncology Group Performance Status (ECOG PS) score (*P* = .001), a high number of metastatic lesions (*P* = .007), and the presence of a primary tumor (*P* = .028), as shown in Table 1. No significant differences related to tumor differentiation, gender, age, and chemotherapy backbones were found between patients with symptoms and patients without symptoms. Among the symptoms at baseline, fatigue was frequently observed (63.6%), and GHS/QOL scores were lower in patients with pain (33.9 ± 5.02) and nausea (33.3 ± 7.93; see Table S2).

**Table 1:** Clinicopathologic correlation with symptom at baseline in 137 metastatic colorectal cancer

Variables	Total No.	Asymptom		Symptom		*P* value
		No.	(%)	No.	(%)	
Total No.	137	82		55		
Age (y)						
Mean ± SEM		65.6 ± 1.1		65.4 ± 1.5		NS (.920)*
<70	86	55	(67.1)	31	(56.4)	NS (.213)
≥70	51	27	(32.9)	24	(43.6)	
GHS/QoL in EORTC QLQ‐C30						**<.0001** *
Mean ± SEM	137	68.1 ± 2.2		49.1 ± 2.9		
Gender						NS (.572)
Male	95	55	(67.1)	40	(72.7)	
Female	42	27	(32.9)	15	(27.3)	
ECOG PS						**.001**
PS0	110	74	(90.2)	36	(65.5)	
PS1 or PS2	27	8	(9.8)	19	(34.5)	
Tumor location						NS (>.999)
Colon	90	54	(65.9)	36	(65.5)	
Rectum	47	28	(34.1)	19	(34.5)	
Differentiation						NS (.267)
Well/mode	129	79	(96.3)	50	(9.9)	
Poor	8	3	(3.7)	5	(9.1)	
Number of metastatic lesions						**.007**
1	51	38	(46.3)	13	(23.6)	
≥2	86	44	(53.7)	42	(76.4)	
Serum CEA (ng/mL)						NS (.184)
<5	25	18	(22.8)	7	(13.2)	
≥5	107	61	(77.2)	46	(86.8)	
Primary tumor						**.028**
Absence	92	61	(74.4)	31	(56.4)	
Presence	45	21	(25.6)	24	(43.6)	
Chemotherapy backbone						NS (.629)
mFOLFOX6	88	54	(65.9)	34	(61.8)	
FOLFIRI	49	28	(34.1)	21	(38.2)	

Abbreviations: ECOG PS, eastern cooperative oncology group performance status; EORTC QLQ‐C30, European Organisation for Research and Treatment of Cancer Quality of Life Questionnaire Core 30; GHS/QoL, global health status/Quality of Life; NS, not significant.

* Unpaired Student's *t* test; the remaining variables, Fisher's exact test.

Bold values indicate statistical significance (*P* < 0.05).

### 3.2 Association of baseline symptoms with prognosis for OS

**Figure 1:**
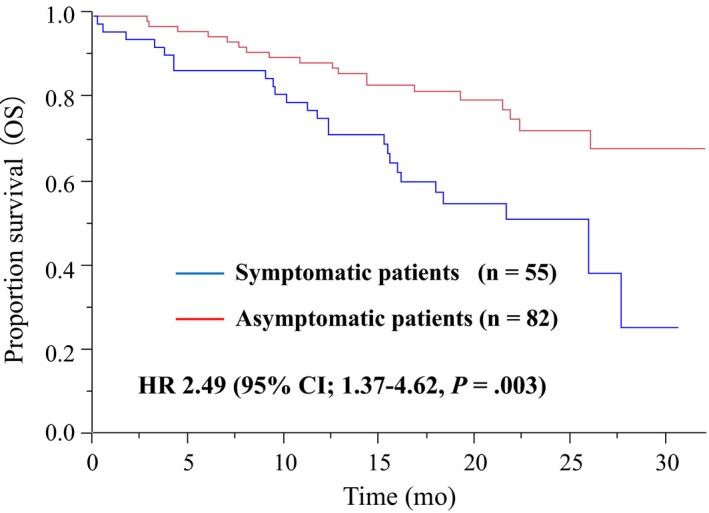
The Kaplan‐Meier curves of OS according to status of baseline symptoms based on the symptom items of EORTC QLQ C‐30 questionnaire in mCRC patients treated with cetuximab plus chemotherapy

The data cutoff date was 20 April 2016, and by that date, 104 and 45 events had been observed in relation to PFS and OS, respectively. The median duration of follow‐up time was 18.0 months (95% CI, 16.5‐19.6), and the median OS had not been reached at the time of the data cutoff. The symptomatic patients at baseline showed significantly worse OS rates compared against asymptomatic patients (HR, 2.49; 95% CI, 1.37‐4.62; *P* = .003; see Figure 1). In addition to baseline symptoms, a patient's age, response to treatment, baseline CEA value, ECOG PS score, and second‐line chemotherapy were factored into prognoses to determine OS rates in univariate analyses (Table 2). Furthermore, baseline symptoms remained an independent predictor of OS (HR, 3.18; 95% CI, 1.48‐6.85; *P* = .003) in the multivariable Cox proportional hazard model with adjustments for these prognostic factors.

**Table 2:** Univariate and multivariable prognostic analyses using the Cox proportional hazard model

Variables	Univariate			Multivariable		
	HR	95% CI	*P* value*	HR	(95% CI)	*P* value*
Symptom at baseline						
Presence vs Absence	2.49	1.37‐4.62	.003	3.18	1.48‐6.85	**.003**
Treatment response						
CR/PR vs SD/PD	0.34	0.18‐0.65	.001	0.33	0.16‐0.67	**.0002**
Second‐line chemotherapy						
Presence vs Absence	0.50	0.74‐2.63	.036	0.43	0.19‐0.98	**.045**
Age						
Age >70 vs <70 (y)	2.07	1.16‐3.73	.014	2.69	1.34‐5.35	**.005**
CEA						
CEA > 5 vs <5	2.07	1.16‐3.73	.014	1.35	0.52‐3.50	.537
ECOG PS						
PS >1 vs PS 0	2.55	1.35‐4.67	.005	1.54	0.59‐4.02	.378
Gender						
Male vs Female	1.34	0.71‐2.70	.373	—	—	—
Chemotherapy backbone				—	—	—
mFOLFOX6 vs FOLFIRI	1.36	0.74‐2.63	.327	—	—	—
Differentiation				—	—	—
Well/mode vs poor	1.09	0.47‐3.15	.856	—	—	—
Tumor location				—	—	—
Colon vs Rectum	1.58	0.30‐1.21	.174	—	—	—
Primary tumor				—	—	—
Presence vs Absence	1.47	0.80‐2.63	.206	—	—	—
Metastatic sites				—	—	—
Liver only vs the other	0.58	0.32‐1.04	.068	—	—	—

Abbreviations: CR, complete response; ECOG PS, eastern cooperative oncology group performance status; PD, progressive disease; PR, partial response; SD, stable disease.

* Cox proportional hazard model.

Bold values indicate statistical significance (*P* < 0.05).

**Figure 2:**
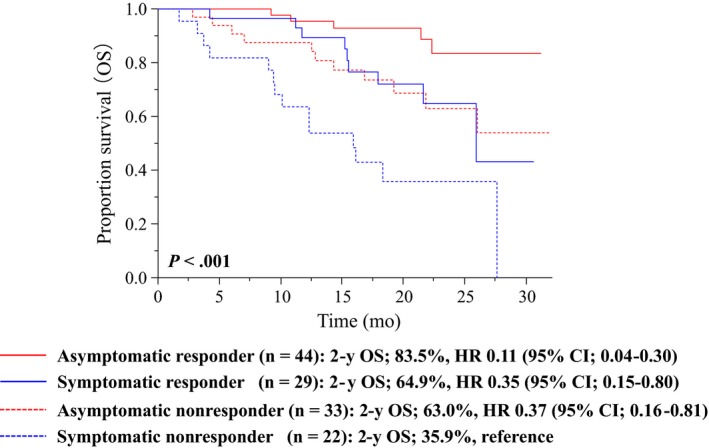
The Kaplan‐Meier curves of OS in combined baseline symptoms and treatment response status. Among asymptomatic patients with data of treatment efficacy (n = 77), 44 and 33 were treatment responders and nonresponder, respectively. Among symptomatic patients (n = 51), 29 and 22 patients were treatment responders and nonresponder, respectively

Our previous study showed that response to treatment improved HRQOL in symptomatic patients.^21^ Since treatment responders had favorable outcomes compared with nonresponders in patients treated with cetuximab plus chemotherapy (Figure S1), the association between baseline symptoms and treatment response was assessed in relation to OS (Figure 2). The asymptomatic responders showed favorable outcomes, whereas the symptomatic nonresponders exhibited worse outcomes (2‐year OS rates: 83.6% for asymptomatic responders and 35.9% for symptomatic nonresponders). The symptomatic responders shared characteristics with asymptomatic nonresponders in relation to OS from a prognostic point of view (2‐year OS rates: 64.9% for symptomatic responders and 63.0% for asymptomatic nonresponders).

### 3.3 Treatment efficacy and AEs in symptomatic patients treated with cetuximab plus chemotherapy

**Figure 3:**
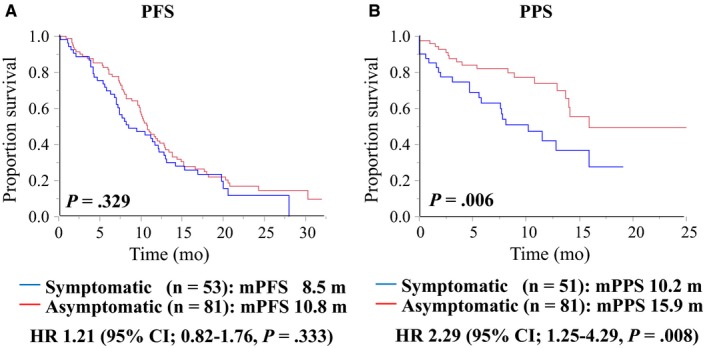
The Kaplan‐Meier curves in terms of (A) progression‐free survival (PFS) and (B) postprogression survival (PPS) according to status of baseline symptoms in mCRC patients treated with cetuximab plus chemotherapy

The efficacy of treatment based on the status of tumor‐related symptoms at baseline was evaluated. The status of baseline symptoms had no significant impact on PFS: The median PFS was 8.5 months (95% CI, 7.0‐12.2) for symptomatic patients and 10.8 months (95% CI, 9.8‐12.8) for asymptomatic patients (log‐rank test, *P* = .329; HR, 1.21; 95% CI, 0.82‐1.76; *P* = .333); see Figure 3A. The objective response rate (ORR) and disease control rate (DCR) were also similar between the patients with and without symptoms (ORR: 52.7% for symptomatic patients and 53.7% for asymptomatic patients; DCR: 85.4% for both groups of patients; see Table S3). Among the symptomatic patients, those with pain had the lowest ORR (30.8%) and DCR (69.2%; see Table S2).

The rate of curative resection of metastases was not significantly different in terms of symptom status (9.8% in asymptomatic patients and 7.3% in symptomatic patients; *P* = .731). Among the patients with disease progression who received first‐line cetuximab plus chemotherapy, second‐line treatment was given to 61 (91.0%) of 67 asymptomatic patients and 32 (74.4%) of 43 symptomatic patients (*P* = .029; see Table S3).

The symptomatic patients had significantly worse outcomes in terms of OS than the asymptomatic patients despite similar PFS and treatment responses. To determine whether the first‐line treatment outcomes reflected the unfavorable OS rates for symptomatic and asymptomatic patients, an exploratory analysis for PPS was conducted (see Figure 3B). The median PPS differed significantly: 10.2 months (95% CI, 5.6‐15.9) for the symptomatic patients compared with 15.9 months (95% CI, 13.7–not reached) for the asymptomatic patients (log‐rank test, *P* = .006), and the corresponding HR was 2.29 (95% CI, 1.25‐4.29, Cox proportional hazard model, *P* = .008).

As described previously, the safety profile in this study was consistent with the results of prior clinical trials.^21^ No significant differences in the overall incidence of grade 3 or higher AEs were observed between the patients with and without symptoms (40% vs 50%; *P* = .295), and both categories of patients also showed similar AE profiles with a grade of ≧ 2 (Table S4). As a cetuximab‐related AE, skin toxicities, including rash, dry skin, acneiform exanthema, paronychia, and pruritus, of grade 2 or higher were observed in 54.5% of the symptomatic patients and 53.7% of the asymptomatic patients.

Finally, the association of functional and GHS/QOL scales at baseline with prognosis, treatment efficacy, and toxicity was analyzed. The response for each scale of a particular dimension was transformed into a score between 0 and 100. These scores are continuous variables and are necessary to dichotomize each score in assessing the association. Therefore, each score was categorized into two groups according to the optimal cutoff value determined by a receiver operating characteristic (ROC) analysis in terms of the presence of symptoms. GHS/QOL and physical functioning were associated with poor OS (Table S5). There were similar objective response rate (ORR), progression‐free survival (PFS), and toxicity profiles for patients irrespective of their functional and GHS/QOL scales. However, these findings and cutoff values are needed to validate.

## 4 DISCUSSION

Traditional endpoints, such as OS and PFS, in clinical trials might not provide either an accurate or a full appreciation of the benefits and risks of therapy from patients’ perspectives in routine clinical practice.^24^ On the other hand, *PROs* refer to a patient's subjective evaluation and satisfaction with treatment in terms of the patient's daily life. Consequently, PROs remain a high‐priority subject in relation to cancer patients and are increasingly recognized as an important component to achieve the paradigm of personalized medicine, not only in routine clinical practice, but also in clinical cancer trials.^13^, ^32^, ^33^ Among the PROs, symptom burden is one of the most relevant dimensions with respect to HRQOL in patients with mCRC.^11^, ^12^ In this regard, a better understanding of its clinical significance may be essential to appropriately manage mCRC. Here, we describe the clinical impact of patient‐reported symptoms at baseline in terms of prognostic relevance, treatment efficacy, and toxicity profiles in the treatment of mCRC with cetuximab plus chemotherapy.

An accurate prognosis has important implications for both patients and physicians because it influences patients’ decisions to undergo chemotherapy and assists patients and families in making the best use of their remaining time together, while for physicians, it guides treatment decisions and supportive care plans. A growing body of evidence suggests that the evaluation of patient‐reported symptoms as a component of HRQOL may be associated with prognosis.^34^, ^35^, ^36^ However, its prognostic value may depend on the specific type of treatment and cancer.^37^ This study validated the association between symptoms at baseline and OS for mCRC patients treated with first‐line cetuximab plus chemotherapy in the CRYSTAL study. Thus, stratifying patients by their baseline symptom burden may be crucial for mCRC patients in clinical trial.

Symptomatic patients at baseline had some clinicopathological features, such as the presence of a primary tumor, more metastatic lesions, and a worsened ECOG PS score, compared with asymptomatic patients. The ECOG PS is a well‐established indicator for prognostic outcomes in cancer patients and was therefore forced into the multivariate model. Note that when it was combined with patient‐reported symptom burden, the prognostic relevance of the ECOG PS was not retained, which is consistent with a previous study.^38^ This indicates that patient‐reported symptoms might provide more useful prognostic information for OS than the patients’ ECOG PS as assessed by physicians despite the significant association between these factors.

Cetuximab has demonstrated efficacy when combined with chemotherapy in the first‐line treatment of left‐sided and RAS wild‐type mCRC.^3^, ^7^ In this study, treatment response showed an improved OS even for symptomatic patients at baseline who were poor prognosis. There were similar toxicity profiles and effects on PFS and ORR for patients receiving cetuximab plus chemotherapy irrespective of their baseline symptom status, indicating that symptom burden is not a negative predictor of treatment efficacy and toxicity. In addition, our previous research on first‐line cetuximab plus chemotherapy showed no deterioration in HRQOL following the addition of cetuximab to chemotherapy (the CRYSTAL study)^39^ and a rapid improvement in HRQOL among the symptomatic patients in terms of treatment response (the QUACK study).^21^ Taken together, cetuximab plus chemotherapy is the most preferred first‐line regimen for left‐sided and RAS wild‐type mCRC with symptoms at baseline from the point of view of HRQOL, treatment efficacy, and safety. In asymptomatic patients, the GHS/QOL score deteriorated to a clinically meaningful degree at 8 weeks in nonresponders, while it was maintained throughout the study period in responders.^21^ Therefore, we will provide information that treatment response maintained HRQOL and prolonged OS for asymptomatic patients. Thus, the routine measurement assessment of patient‐reported symptoms may provide additional information to facilitate personalized decision‐making which includes the patient's perspective and the proper management in clinical practice.

Despite the similar treatment efficacy of cetuximab plus chemotherapy, statistically different PPS among those patients with and without symptoms was observed. Although several effective agents are available for later‐line chemotherapy for mCRC, approximately 50%‐60% of patients starting first‐line chemotherapy receive a next‐line therapy.^40^ Therefore, the frequency of second‐line treatment administered after disease progression following first‐line chemotherapy may influence PPS and, subsequently, OS.^25^ In the present study, 91.0% of the patients who were asymptomatic at baseline for first‐line therapy received second‐line treatment, while 74.4% of the patients who were symptomatic at baseline for first‐line therapy received subsequent chemotherapy. However, the baseline symptom burden remained an independent prognostic predictor even after adjusting for administration (yes vs no) of second‐line treatment (Table 2). Patient‐reported symptoms at baseline may therefore be a surrogate marker of the biological roles in tumor behavior following treatment with cetuximab plus chemotherapy.

It is very important to show the difference between patient‐ and physician‐reported outcomes at baseline. As a decision is often made on whether to treat or reduce the dose when nonhematologic adverse events (AEs) are NCI‐CTCAE grade 2 or higher in clinical trials, the inclusion criteria included patients with grade 0 or 1 nonhematologic toxicity at baseline by the NCI‐CTCAE version 4.0 in this study. Physicians reported no patients with clinically significant symptoms at baseline, and eight symptoms (nausea/vomiting, fatigue, constipation, dyspnea, pain, appetite loss, diarrhea, and insomnia) of the EORTC QLQ‐C30 questionnaire were classified as grade 0 according to the NCI‐CTCAE version 4.0 for almost all patients. Nevertheless, 55 of 137 patients (40%) reported “quite a bit” or “very much” to at least one of the eight single‐item symptom questions by the EORTC QLQ‐C30. In fact, the GHS/QOL scales were statistically higher in the asymptomatic patients compared with the symptomatic patients (68.1 for asymptomatic patients, and 49.1 for symptomatic patients; *P* < .0001, Table 1), and the difference (Δ19 points) was clinically relevant.^30^ The NCI‐CTCAE is generally used to assess any abnormal clinical findings temporally associated with the use of a medical treatment, but not the cancer itself, by physicians.^41^, ^42^ The EORTC QLQ‐C30 is used to assess patients’ QoL status by patient self‐reports, independent of a medical treatment. Thus, the NCI‐CTCAE and EORTC QLQ‐C30 were intended for different purposes, and the conceptual and methodological differences might result in different perspectives reported by patients and physicians at baseline.^42^ These findings may imply that patient‐reported symptoms capture the global status of patients’ subjective perspectives, physical health, and tumor burden. As the status of baseline symptoms is easily categorized into two groups using the symptom questionnaire of the EORTC QLQ‐C30, this simple, timely, and cost‐effective method is useful for assessment of baseline symptoms and prognostic estimations in clinical practice.

The main limitation of the present study was the relatively small sample size, and part of the statistical analyses were deemed unreliable. In some instances, specific symptoms were likely to have prognostic value: for example, dysphagia in esophageal cancer,^35^ loss of appetite in breast cancer and in CRC,^36^, ^43^ and pain in lung cancer and in CRC.^34^, ^44^ In addition, mucositis/stomatitis was the most substantial AE compromising both QOL and treatment compliance in CRC, as reported previously.^21^ However, it is unclear which symptoms had the greatest impact on OS outcomes when treatment used cetuximab plus chemotherapy due to the small sample size. It is also unclear how differences in racial, physical backgrounds, and ethnic affect the perceptions of symptoms, since the study focused on the Japanese population. Clinical trials usually exclude patients with comorbidities, patients with a severe symptom burden, and elderly patients at the time of study entry, thus limiting trials to relatively asymptomatic populations. In fact, most patients in our study had a good performance status (79.3% for ECOG PS0). Primary tumors arising from different sides of the colon (left versus right) have different clinical outcomes for anti‐EGFR ab plus chemotherapy; patients with RAS wild‐type and left‐sided mCRC benefit more from treatment with cetuximab plus chemotherapy.^7^ However, at the time of starting this study, the concept of “tumor sidedness for treatment with anti‐EGFR ab” had not yet been established in clinical practice. Therefore, we had not collected data about location of the primary tumor in this study. As the presence of symptoms was one of the poor prognostic factors independent of treatment response for cetuximab plus chemotherapy, in multivariate analysis for OS (Table 2), it is possible that baseline symptoms may be associated with worse OS even for patients with RAS wild‐type and left‐sided mCRC in treatment with first‐line cetuximab plus chemotherapy. Future studies according to tumor location are needed.

Finally, post hoc analysis was performed using prospective study data and should therefore be considered as hypothesis generation. On the other hand, the main strengths of this study include the use of data from a prospectively designed study for QOL with a high rate of questionnaire completion using the well‐established global EORTC QLQ‐C30. In addition, all patients received cetuximab plus chemotherapy, indicating a homogenous population. Future studies are needed to confirm the findings of this study.

In conclusion, the routine measurement assessment of patient‐reported symptoms before starting treatment in clinical practice may be useful for patients and physicians to make more properly informed treatment decisions.

## ETHICS APPROVAL AND CONSENT TO PARTICIPATE

This study has been conducted according to the criteria set by the Declaration of Helsinki and the Ethics Guidelines for Clinical Research by the Ministry of Health, Labor, and Welfare in Japan. Written Informed consent was obtained from all patients before the start of study treatment. The study design was approved by the ethics committee or institutional review board of each participating institution. It was registered with the University Hospital Medical Information Network (UMIN) Clinical Trial Registry (UMIN000010985) on July 19, 2013.

## Data Availability

The data that support the findings of this study are available from the corresponding author upon reasonable request.

## References

[1] Siegel RL , Miller KD , Fedewa SA , et al. Colorectal cancer statistics, 2017. CA Cancer J Clin. 2017;67:177‐193. 28248415 10.3322/caac.21395

[2] Van Cutsem E , Cervantes A , Nordlinger B , Arnold D . Metastatic colorectal cancer: ESMO Clinical Practice Guidelines for diagnosis, treatment and follow‐up. Ann Oncol. 2014;25(suppl 3):iii1‐iii9. 25190710 10.1093/annonc/mdu260

[3] NCCN Clinical Practice Guidelines in Oncology, Colon Cancer. 2018; version 4. http://www.nccn.org/professionals/physician_gls/f_guidelines.asp.

[4] Au HJ , Karapetis CS , O'Callaghan CJ , et al. Health‐related quality of life in patients with advanced colorectal cancer treated with cetuximab: overall and KRAS‐specific results of the NCIC CTG and AGITG CO.17 Trial. J Clin Oncol. 2009;27:1822‐1828. 19273701 10.1200/JCO.2008.19.6048

[5] Arnold D , Lueza B , Douillard JY , et al. Prognostic and predictive value of primary tumour side in patients with RAS wild‐type metastatic colorectal cancer treated with chemotherapy and EGFR directed antibodies in six randomized trials. Ann Oncol. 2017;28:1713‐1729. 28407110 10.1093/annonc/mdx175 PMC6246616

[6] Tejpar S , Stintzing S , Ciardiello F , et al. Prognostic and predictive relevance of primary tumor location in patients with RAS wild‐type metastatic colorectal cancer: retrospective analyses of the CRYSTAL and FIRE‐3 Trials. JAMA Oncol. 2017;3:194‐201. 27722750 10.1001/jamaoncol.2016.3797 PMC7505121

[7] Yoshino T , Arnold D , Taniguchi H , et al. Pan‐Asian adapted ESMO consensus guidelines for the management of patients with metastatic colorectal cancer: a JSMO‐ESMO initiative endorsed by CSCO, KACO, MOS, SSO and TOS. Ann Oncol. 2018;29:44‐70. 29155929 10.1093/annonc/mdx738

[8] Van Cutsem E , Cervantes A , Adam R , et al. ESMO consensus guidelines for the management of patients with metastatic colorectal cancer. Ann Oncol. 2016;27:1386‐1422. 27380959 10.1093/annonc/mdw235

[9] van Leeuwen M , Husson O , Alberti P , et al. Understanding the quality of life (QOL) issues in survivors of cancer: towards the development of an EORTC QOL cancer survivorship questionnaire. Health Qual Life Outcomes. 2018;16:114. 29866185 10.1186/s12955-018-0920-0 PMC5987570

[10] Ramsey SD , Berry K , Moinpour C , Giedzinska A , Andersen MR . Quality of life in long term survivors of colorectal cancer. Am J Gastroenterol. 2002;97:1228‐1234. 12017152 10.1111/j.1572-0241.2002.05694.x

[11] Gray NM , Hall SJ , Browne S , et al. Modifiable and fixed factors predicting quality of life in people with colorectal cancer. Br J Cancer. 2011;104:1697‐1703. 21559017 10.1038/bjc.2011.155 PMC3111166

[12] Marventano S , Forjaz M , Grosso G , et al. Health related quality of life in colorectal cancer patients: state of the art. BMC Surg. 2013;13(Suppl 2):S15. 24267735 10.1186/1471-2482-13-S2-S15 PMC3851259

[13] Secord AA , Coleman RL , Havrilesky LJ , Abernethy AP , Samsa GP , Cella D . Patient‐reported outcomes as end points and outcome indicators in solid tumours. Nat Rev Clin Oncol. 2015;12:358‐370. 25754949 10.1038/nrclinonc.2015.29

[14] Basch E , Iasonos A , McDonough T , et al. Patient versus clinician symptom reporting using the National Cancer Institute Common Terminology Criteria for Adverse Events: results of a questionnaire‐based study. Lancet Oncol. 2006;7:903‐909. 17081915 10.1016/S1470-2045(06)70910-X

[15] Basch E , Dueck AC , Rogak LJ , et al. Feasibility assessment of patient reporting of symptomatic adverse events in multicenter cancer clinical trials. JAMA Oncol. 2017;3:1043‐1050. 28208174 10.1001/jamaoncol.2016.6749 PMC5553624

[16] Gotay CC , Kawamoto CT , Bottomley A , Efficace F . The prognostic significance of patient‐reported outcomes in cancer clinical trials. J Clin Oncol. 2008;26:1355‐1363. 18227528 10.1200/JCO.2007.13.3439

[17] Quinten C , Coens C , Mauer M , et al. Baseline quality of life as a prognostic indicator of survival: a meta‐analysis of individual patient data from EORTC clinical trials. Lancet Oncol. 2009;10:865‐871. 19695956 10.1016/S1470-2045(09)70200-1

[18] Efficace F , Innominato PF , Bjarnason G , et al. Validation of patient's self‐reported social functioning as an independent prognostic factor for survival in metastatic colorectal cancer patients: results of an international study by the Chronotherapy Group of the European Organisation for Research and Treatment of Cancer. J Clin Oncol. 2008;26:2020‐2026. 18421055 10.1200/JCO.2007.12.3117

[19] Lee CK , Stockler MR , Coates AS , Gebski V , Lord SJ , Simes RJ . Self‐reported health‐related quality of life is an independent predictor of chemotherapy treatment benefit and toxicity in women with advanced breast cancer. Br J Cancer. 2010;102:1341‐1347. 20389302 10.1038/sj.bjc.6605649 PMC2865758

[20] Ooki A , Ando M , Sakamoto J , Sato A , Fujii H , Yamaguchi K . A prospective observational study to examine the relationship between quality of life and adverse events of first‐line chemotherapy plus cetuximab in patients with KRAS wild‐type unresectable metastatic colorectal cancer: QUACK Trial. Jpn J Clin Oncol. 2014;44:383‐387. 24558128 10.1093/jjco/hyu008

[21] Iwamoto S , Ooki A , Morita S , et al. A prospective Phase II study to examine the relationship between quality of life and adverse events of first‐line chemotherapy plus cetuximab in patients with KRAS wild‐type unresectable metastatic colorectal cancer: QUACK trial. Cancer Med. 2018;7:4217‐4227. 30051609 10.1002/cam4.1623 PMC6144158

[22] Aaronson NK , Ahmedzai S , Bergman B , et al. The European Organization for Research and Treatment of Cancer QLQ‐C30: a quality‐of‐life instrument for use in international clinical trials in oncology. J Natl Cancer Inst. 1993;85:365‐376. 8433390 10.1093/jnci/85.5.365

[23] Byrne C , Griffin A , Blazeby J , Conroy T , Efficace F . Health‐related quality of life as a valid outcome in the treatment of advanced colorectal cancer. Eur J Surg Oncol. 2007;33(Suppl 2):S95‐S104. 18039559 10.1016/j.ejso.2007.10.003

[24] Bonnetain F , Borg C , Adams RR , et al. How health‐related quality of life assessment should be used in advanced colorectal cancer clinical trials. Ann Oncol. 2017;28:2077‐2085. 28430862 10.1093/annonc/mdx191

[25] Petrelli F , Barni S . Correlation of progression‐free and post‐progression survival with overall survival in advanced colorectal cancer. Ann Oncol. 2013;24:186‐192. 22898038 10.1093/annonc/mds289

[26] Van Cutsem E , Kohne CH , Hitre E , et al. Cetuximab and chemotherapy as initial treatment for metastatic colorectal cancer. N Engl J Med. 2009;360:1408‐1417. 19339720 10.1056/NEJMoa0805019

[27] Heinemann V , von Weikersthal LF , Decker T , et al. FOLFIRI plus cetuximab versus FOLFIRI plus bevacizumab as first‐line treatment for patients with metastatic colorectal cancer (FIRE‐3): a randomised, open‐label, phase 3 trial. Lancet Oncol. 2014;15:1065‐1075. 25088940 10.1016/S1470-2045(14)70330-4

[28] Potthoff K , Hofheinz R , Hassel JC , et al. Interdisciplinary management of EGFR‐inhibitor‐induced skin reactions: a German expert opinion. Ann Oncol. 2011;22:524‐535. 20709812 10.1093/annonc/mdq387

[29] Fayers P , Aaronson NK , Bjordal K . The EORTC QLQ‐C30 Scoring Manual (ed. 2). Brussels, Belgium: Eurpoean Organisation for Research and Treatment of Cancer; 1999.

[30] Osoba D , Rodrigues G , Myles J , Zee B , Pater J . Interpreting the significance of changes in health‐related quality‐of‐life scores. J Clin Oncol. 1998;16:139‐144. 9440735 10.1200/JCO.1998.16.1.139

[31] Lang I , Kohne CH , Folprecht G , et al. Quality of life analysis in patients with KRAS wild‐type metastatic colorectal cancer treated first‐line with cetuximab plus irinotecan, fluorouracil and leucovorin. Eur J Cancer. 2013;49:439‐448. 23116683 10.1016/j.ejca.2012.08.023

[32] Vodicka E , Kim K , Devine EB , Gnanasakthy A , Scoggins JF , Patrick DL . Inclusion of patient‐reported outcome measures in registered clinical trials: Evidence from ClinicalTrials.gov (2007–2013). Contemp Clin Trials. 2015;43:1‐9. 25896116 10.1016/j.cct.2015.04.004

[33] Fiteni F , Pam A , Anota A , et al. Health‐related quality‐of‐life as co‐primary endpoint in randomized clinical trials in oncology. Expert Rev Anticancer Ther. 2015;15:885‐891. 26027598 10.1586/14737140.2015.1047768

[34] Ediebah DE , Coens C , Zikos E , et al. Does change in health‐related quality of life score predict survival? Analysis of EORTC 08975 lung cancer trial. Br J Cancer. 2014;110:2427‐2433. 24743709 10.1038/bjc.2014.208 PMC4021536

[35] Fang FM , Tsai WL , Chiu HC , Kuo WR , Hsiung CY . Quality of life as a survival predictor for esophageal squamous cell carcinoma treated with radiotherapy. Int J Radiat Oncol Biol Phys. 2004;58:1394‐1404. 15050315 10.1016/j.ijrobp.2003.09.100

[36] Efficace F , Biganzoli L , Piccart M , et al. Baseline health‐related quality‐of‐life data as prognostic factors in a phase III multicentre study of women with metastatic breast cancer. Eur J Cancer. 2004;40:1021‐1030. 15093577 10.1016/j.ejca.2004.01.014

[37] Quinten C , Martinelli F , Coens C , et al. A global analysis of multitrial data investigating quality of life and symptoms as prognostic factors for survival in different tumor sites. Cancer. 2014;120:302‐311. 24127333 10.1002/cncr.28382

[38] Maisey NR , Norman A , Watson M , Allen MJ , Hill ME , Cunningham D . Baseline quality of life predicts survival in patients with advanced colorectal cancer. Eur J Cancer. 2002;38:1351‐1357. 12091066 10.1016/s0959-8049(02)00098-9

[39] Yamaguchi K , Ando M , Ooki A , et al. Quality of life analysis in patients with RAS wild‐type metastatic colorectal cancer treated with first‐line cetuximab plus chemotherapy. Clin Colorectal Cancer. 2017;16(2):e29‐e37. 28081962 10.1016/j.clcc.2016.07.017

[40] Grothey A , Sargent D . Overall survival of patients with advanced colorectal cancer correlates with availability of fluorouracil, irinotecan, and oxaliplatin regardless of whether doublet or single‐agent therapy is used first line. J Clin Oncol. 2005;23:9441‐9442. 16361649 10.1200/JCO.2005.04.4792

[41] National Cancer Institute . Common terminology criteria for adverse events (CTCAE) Version 4.0. https://ctep.cancer.gov/protocolDevelopment/electronic_applications/ctc.htm#ctc_40; 2010.

[42] Quinten C , Maringwa J , Gotay CC , et al. Patient self‐reports of symptoms and clinician ratings as predictors of overall cancer survival. J Natl Cancer Inst. 2011;103:1851‐1858. 22157640 10.1093/jnci/djr485 PMC3243678

[43] Braun DP , Gupta D , Grutsch JF , Staren ED . Can changes in health related quality of life scores predict survival in stages III and IV colorectal cancer? Health Qual Life Outcomes. 2011;9:62. 21812962 10.1186/1477-7525-9-62 PMC3162879

[44] Diouf M , Chibaudel B , Filleron T , et al. Could baseline health‐related quality of life (QoL) predict overall survival in metastatic colorectal cancer? The results of the GERCOR OPTIMOX 1 study. Health Qual Life Outcomes. 2014;12:69. 24886671 10.1186/1477-7525-12-69 PMC4029890

