# Supplementary figures and images for: Patient‐reported symptom burden as a prognostic factor in treatment with first‐line cetuximab plus chemotherapy for unresectable metastatic colorectal cancer: Results of Phase II QUACK trial
Source: Cancer Med. 2020 Jan 21;9(5):1779–89. doi: 10.1002/cam4.2826 (PMC7050093; doi:10.1002/cam4.2826)

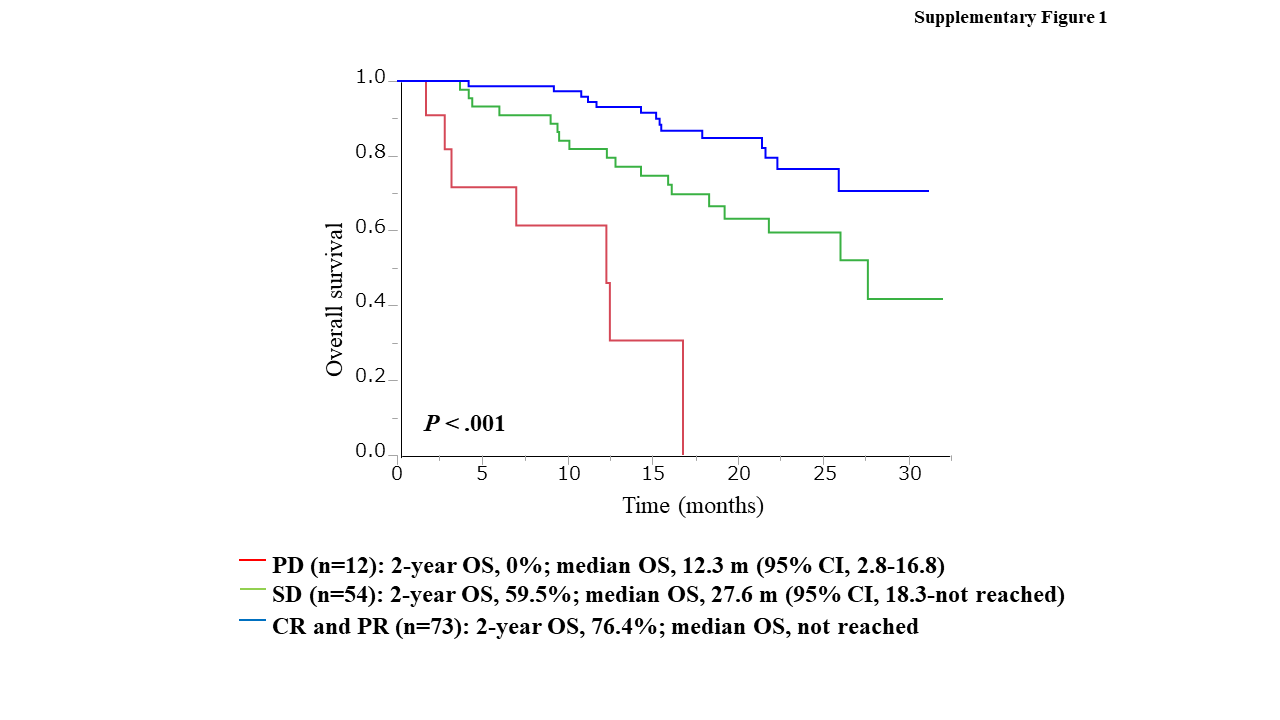

Supplement: Supplementary file 1 [file CAM4-9-1779-s001.tif]
