# Supplementary material for: Patient‐reported symptom burden as a prognostic factor in treatment with first‐line cetuximab plus chemotherapy for unresectable metastatic colorectal cancer: Results of Phase II QUACK trial
Source: Cancer Med. 2020 Jan 21;9(5):1779–89. doi: 10.1002/cam4.2826 (PMC7050093; doi:10.1002/cam4.2826)
Supplement: Supplementary file 7 [file CAM4-9-1779-s007.docx]

**Appendix**:

| **Supplementary Table S1: Participating institutions and principal investigators in QUACK study** |
| --- |
| **Site Name** |
| Kobe City Medical Center General Hospital |
| Saitama Cancer Center |
| Kansai Medical University Hospital |
| Okayama Rosai Hospital |
| National Hospital Organization Nagoya Medical Center |
| Kansai Rosai Hospital |
| Sano Hospital |
| Aizawa Hospital |
| Osaka Saiseikai Senri Hospital |
| Yamagata Prefectural Central Hospital |
| Nishinomiya Municipal Central Hospital |
| Shikoku Central Hospital |
| Teikyo University Hospital |
| Yokohama Municipal Citizen's Hospital |
| Tokai Central Hospital |
| Gifu University Hospital |
| Japanese Red Cross Medical Center |
| Ina Central Hospital |
| Keiyukai Sapporo Hospital |
| Kiyoukai Okamoto Hospital |
| Kyoto Miniren Chuo Hospital |
| Fukuiken Saiseikai Hospital |
| National Hospital Organization Kobe Medical Center |
| Matsuyama Red Cross Hospital |
| Higashiosaka City General Hospital |
| Machida Municipal Hospital |
| Kansai Medical University Takii Hospital |
| Matsuda Hospital |
| Gifu Prefectural Tajimi Hospital |
| Japan Community Health care Organization Kanazawa Hospital |
| Japan Post Kyoto Teishin Hospital |
| Ogaki Municipal Hospital |
| Saiseikai Shigaken Hospital |
| Kanazawa Medical University |
| Matsushita Memorial Hospital |
| Kyoto Prefectural University of Medicine |
| Kinki Central Hospital of the Mutual Aid Association of Public School Teachers |
| Kawasaki Hospital |
| Kaizuka City Hospital |
| Tokyo Metropolitan Tama Medical Center |
| Kikugawa General Hospital |
| Hirakata kohsai Hospital |
| Kouseiren Takaoka Hospital |
| Tachikawa Medical Center |
| Japanese Red Cross Otsu Hospital |
| Kagawa University Hospital |
| National Hospital Organization Fukuyama Medical Center |
| Takarazuka City Hospital |
| Kansai Medical University Kori Hospital |

| **Supplementary Table S2. Features of each symptom item in symptomatic patients at baseline** | | | | | | | | |
| --- | --- | --- | --- | --- | --- | --- | --- | --- |
| **Symptom items (n=55)** |  | **No. of patients (%)** |  | **GHS score (Mean + SEM)** |  | **No. of response (%)** |  | **No. of disease control (%)** |
| **Symptom** |  | 55 (100) |  | 49.1 + 2.87 |  | 29 (55.8) |  | 47 (90.4) |
| Fatigue |  | 35 (63.6) |  | 48.0 + 3.76 |  | 12 (34.3) |  | 31 (88.6) |
| Appetite loss |  | 18 (32.7) |  | 35.2 + 4.98 |  | 7 (43.8) |  | 13 (81.3) |
| Pain |  | 16 (29.1) |  | 33.9 + 5.02 |  | 4 (30.8) |  | 9 (69.2) |
| Constipation |  | 14 (25.5) |  | 48.2 + 4.21 |  | 9 (64.3) |  | 14 (100) |
| Sleep disturbance |  | 13 (23.6) |  | 38.5 + 7.10 |  | 6 (54.5) |  | 10 (90.9) |
| Diarrhea |  | 10 (18.2) |  | 35.8 + 7.56 |  | 4 (44.4) |  | 8 (88.9) |
| Nausea |  | 7 (12.7) |  | 33.3 + 7.93 |  | 3 (42.9) |  | 5 (71.4) |
| Dyspnea |  | 6 (10.9) |  | 34.7 + 9.96 |  | 3 (50.0) |  | 5 (83.3) |

**Abbreviations**: No., number; SEM, standard error of mean; GHS, global health status.

Each symptom was defined as positive if patients answered ‘quite a bit’ or ‘very much’ to at least one of the indicated symptom questions of EORTC QLQ-C30 at baseline.

| **Supplementary Table S3. Treatment efficacy according to the symptom status** | | | | | | |
| --- | --- | --- | --- | --- | --- | --- |
| **Treatment efficacy** |  | **Asymptomatic patients (n=82)** |  | **Symptomatic patients (n=55)** |  | ***P* value** |
|  |  | **Number of patients (%)** |  | **Number of patients (%)** |  |  |
| Best overall response |  |  |  |  |  | NS (>.999)* |
| CR/PR |  | 44 (53.7) |  | 29 (52.7) |  | NS (>.999)** |
| SD |  | 26 (31.7) |  | 18 (32.7) |  |  |
| PD |  | 7 (8.5) |  | 5 (9.1) |  |  |
| NE |  | 5 (6.1) |  | 3 (5.5) |  |  |
| No treatment after curative surgery |  | 8 (9.8) |  | 4 (7.3) |  | NS (.731) |
| Second-line chemotherapy |  |  |  |  |  | **.029** |
| Absence |  | 6 (9.0) |  | 11 (25.6) |  |  |
| Presence |  | 61 (91.0) |  | 32 (74.4) |  |  |
| On treatment of first-line |  | 7 (8.5) |  | 6 (10.9) |  |  |
| Missing |  | 0 (0.0) |  | 1 (1.8) |  |  |

**Abbreviations**: CR, complete response; PR, partial response; SD, stable disease; PD, progressive disease; NE, not evaluable; NS, not significant.

* CR/PR vs. SD/PD, ** CR/PR/SD vs. PD. Fisher's exact test was performed.

The others included missing data, no recurrence after curative surgery (n=12), and on treatment of first line chemotherapy

| **Supplementary Table S4. Correlation between adverse events and symptom at baseline** | | | | | | | | | |
| --- | --- | --- | --- | --- | --- | --- | --- | --- | --- |
|  | | | | | | | | |  |
| **Variables** | **Total No.** |  | **Asymptomatic patients** | |  | **Symptomatic patients** | |  | ***P* value** |
|  |  |  | **No.** | **(%)** |  | **No.** | **(%)** |  |  |
| Total No. | 137 |  | 82 | |  | 55 | |  |  |
| Skin reaction |  |  |  |  |  |  |  |  | NS (>.999) |
| Grade 0/1 | 63 |  | 38 | (46.3) |  | 25 | (45.5) |  |  |
| Grade > 2 | 74 |  | 44 | (53.7) |  | 30 | (54.5) |  |  |
| Hepatic dysfunction |  |  |  |  |  |  |  |  | **.032** |
| Grade 0/1 | 116 |  | 74 | (90.2) |  | 42 | (76.4) |  |  |
| Grade > 2 | 21 |  | 8 | (9.8) |  | 13 | (23.6) |  |  |
| Renal dysfunction |  |  |  |  |  |  |  |  | NS (>.999) |
| Grade 0/1 | 123 |  | 74 | (90.2) |  | 49 | (89.8) |  |  |
| Grade > 2 | 14 |  | 8 | (9.8) |  | 6 | (10.2) |  |  |
| Electrolyte imbalance |  |  |  |  |  |  |  |  | NS (.220) |
| Grade 0/1 | 105 |  | 66 | (80.5) |  | 39 | (70.9) |  |  |
| Grade > 2 | 32 |  | 16 | (19.5) |  | 16 | (29.1) |  |  |
| Neutropenia |  |  |  |  |  |  |  |  | NS (.807) |
| Grade 0/1 | 69 |  | 42 | (51.2) |  | 27 | (49.1) |  |  |
| Grade > 2 | 68 |  | 40 | (48.8) |  | 28 | (50.9) |  |  |
| Anemia |  |  |  |  |  |  |  |  | NS (.284) |
| Grade 0/1 | 50 |  | 33 | (40.2) |  | 17 | (30.9) |  |  |
| Grade > 2 | 87 |  | 49 | (59.8) |  | 38 | (69.1) |  |  |
| Nausea |  |  |  |  |  |  |  |  | NS (>.999) |
| Grade 0/1 | 117 |  | 70 | (85.4) |  | 47 | (85.4) |  |  |
| Grade > 2 | 20 |  | 12 | (14.6) |  | 8 | (14.6) |  |  |
| Vomotting |  |  |  |  |  |  |  |  | NS (.484) |
| Grade 0/1 | 128 |  | 78 | (95.1) |  | 50 | (90.9) |  |  |
| Grade > 2 | 9 |  | 4 | (4.9) |  | 5 | (9.1) |  |  |
| Diarrhea |  |  |  |  |  |  |  |  | NS (>.999) |
| Grade 0/1 | 123 |  | 74 | (90.2) |  | 49 | (89.1) |  |  |
| Grade > 2 | 14 |  | 8 | (9.8) |  | 6 | (10.9) |  |  |
| Fatigue |  |  |  |  |  |  |  |  | NS (.414) |
| Grade 0/1 | 105 |  | 65 | (79.3) |  | 40 | (72.7) |  |  |
| Grade > 2 | 32 |  | 17 | (20.7) |  | 15 | (27.3) |  |  |
| Mucositis/stomatitis |  |  |  |  |  |  |  |  | **.019** |
| Grade 0/1 | 114 |  | 63 | (76.8) |  | 51 | (92.7) |  |  |
| Grade > 2 | 23 |  | 19 | (23.2) |  | 4 | (7.3) |  |  |
| Anorexia |  |  |  |  |  |  |  |  | NS (>.999) |
| Grade 0/1 | 104 |  | 62 | (75.6) |  | 42 | (76.4) |  |  |
| Grade > 2 | 33 |  | 20 | (24.4) |  | 13 | (23.6) |  |  |
| Alopecia |  |  |  |  |  |  |  |  | NS (.702) |
| Grade 0/1 | 130 |  | 77 | (93.9) |  | 53 | (96.4) |  |  |
| Grade > 2 | 7 |  | 5 | (6.1) |  | 2 | (3.6) |  |  |
| Constipation |  |  |  |  |  |  |  |  | NS (.242) |
| Grade 0/1 | 124 |  | 72 | (87.8) |  | 52 | (94.5) |  |  |
| Grade > 2 | 13 |  | 10 | (12.2) |  | 3 | (5.5) |  |  |

Adverse events severity was graded according to National Cancer Institute’s Common Toxicity Criteria version 4.0. NS, not significant. Fisher's exact test was performed

| **Supplementary Table S5. The association of functional and GHS/QOL scales with prognosis, therapeutic efficacy, and toxicity** | | | | | | | | | | | | | |
| --- | --- | --- | --- | --- | --- | --- | --- | --- | --- | --- | --- | --- | --- |
| **Treatment efficacy** |  | **GHS/QOL (cut-off value = 58.3 )** | | |  | | **Physical (cut-off value = 86.7 )** | | |  | **Role (cut-off value = 83.3 )** | | |
|  |  | **High** | **Low** | ***P* value** |  | | **High** | **Low** | ***P* value** |  | **High** | **Low** | ***P* value** |
| Total No. (%) |  | 73 (53.3) | 64 (46.7) |  |  | | 90 (66.2) | 46 (33.8) |  |  | 97 (73.5) | 35 (26.5) |  |
| 2 year-OS (%) |  | 71.5 | 56.6 | **.047*** |  | | 71.5 | 52.1 | **.004*** |  | 64.1 | 65.5 | NS (.333)* |
| mPFS (months) |  | 11.3 | 10.3 | NS (.559)* |  | | 10.8 | 8.4 | NS (.689)* |  | 10.4 | 11.5 | NS (.739)* |
| 95% CI |  | 8.5-12.9 | 7.7-11.7 |  |  | | 9.6-12.2 | 7.2-13.8 |  |  | 8.9-12.0 | 5.6-15.2 |  |
| Best overall response: No. (%) |  |  |  | NS (.256)** |  | |  |  | NS (>.999)** |  |  |  | NS (>.999)** |
| CR/PR |  | 37 (50.7) | 36 (56.2) | NS (.713)*** |  | | 49 (54.4) | 23 (50.0) | NS (.197)*** |  | 52 (53.6) | 18 (51.4) | **.033***** |
| SD |  | 28 (38.4) | 16 (25.0) |  |  | | 32 (35.6) | 12 (26.1) |  |  | 35 (36.1) | 8 (22.9) |  |
| PD |  | 6 (8.2) | 6 (9.4) |  |  | | 6 (6.7) | 6 (13.0) |  |  | 5 (5.2) | 6 (17.1) |  |
| NE |  | 2 (2.7) | 6 (9.4) |  |  | | 3 (3.3) | 5 (10.9) |  |  | 5 (5.2) | 3 (8.6) |  |
| Adverse events: No. (%) |  |  |  |  |  | |  |  |  |  |  |  |  |
| Skin reaction |  |  |  | NS (.864) |  | |  |  | NS (.585) |  |  |  | NS (>.999) |
| Grade 0/1 |  | 34 (46.6) | 28 (44.4) |  |  | | 39 (43.3) | 22 (48.9) |  |  | 44 (45.4) | 15 (44.1) |  |
| Grade > 2 |  | 39 (53.4) | 35 (55.6) |  |  | | 51 (56.7) | 23 (51.1) |  |  | 53 (54.6) | 19 (55.9) |  |
| Hepatic dysfunction |  |  |  | NS (.347) |  | |  |  | NS (.307) |  |  |  | NS (.055) |
| Grade 0/1 |  | 64 (87.7) | 52 (81.3) |  |  | | 79 (87.8) | 37 (80.4) |  |  | 86 (88.7) | 26 (74.3) |  |
| Grade > 2 |  | 9 (12.3) | 12 (18.7) |  |  | | 11 (12.2) | 9 (19.6) |  |  | 11 (11.3) | 9 (25.7) |  |
| Renal dysfunction |  |  |  | NS (>.999) |  | |  |  | NS (.363) |  |  |  | NS (.515) |
| Grade 0/1 |  | 66 (90.4) | 57 (89.1) |  |  | | 83 (92.2) | 40 (87.0) |  |  | 87 (89.7) | 33 (94.3) |  |
| Grade > 2 |  | 7 (9.6) | 7 (10.9) |  |  | | 7 (7.8) | 6 (13.0) |  |  | 10 (10.3) | 2 (5.7) |  |
| Electrolyte imbalance |  |  |  | **0.046** |  | |  |  | **0.029** |  |  |  | **0.009** |
| Grade 0/1 |  | 61 (83.6) | 44 (68.8) |  |  | | 75 (83.3) | 30 (65.2) |  |  | 81 (83.5) | 21 (60.0) |  |
| Grade > 2 |  | 12 (16.4) | 20 (31.2) |  |  | | 15 (16.7) | 16 (34.8) |  |  | 16 (16.5) | 14 (40.0) |  |
| Neutropenia |  |  |  | NS (.172) |  | |  |  | NS (.857) |  |  |  | NS (.844) |
| Grade 0/1 |  | 41 (56.4) | 28 (43.8) |  |  | | 45 (50.0) | 24 (52.2) |  |  | 49 (50.5) | 19 (54.3) |  |
| Grade > 2 |  | 32 (43.8) | 36 (56.2) |  |  | | 45 (50.0) | 22 (47.8) |  |  | 48 (49.5) | 16 (45.7) |  |
| Anemia |  |  |  | NS (.155) |  | |  |  | NS (.348) |  |  |  | NS (.105) |
| Grade 0/1 |  | 31 (42.5) | 19 (29.7) |  |  | | 36 (40.0) | 14 (30.4) |  |  | 41 (42.3) | 9 (25.7) |  |
| Grade > 2 |  | 42 (57.5) | 45 (70.3) |  |  | | 54 (60.0) | 32 (69.6) |  |  | 56 (57.7) | 26 (74.3) |  |
| Nausea |  |  |  | NS (.092) |  | |  |  | NS (.308) |  |  |  | NS (.170) |
| Grade 0/1 |  | 66 (90.4) | 51 (79.7) |  |  | | 79 (87.8) | 37 (80.4) |  |  | 85 (87.6) | 27 (77.1) |  |
| Grade > 2 |  | 7 (9.6) | 13 (20.3) |  |  | | 11 (12.2) | 9 (19.6) |  |  | 12 (12.4) | 8 (22.9) |  |
| Vomiting |  |  |  | **0.012** |  | |  |  | NS (.166) |  |  |  | NS (.208) |
| Grade 0/1 |  | 72 (98.6) | 56 (87.5) |  |  | | 86 (95.6) | 41 (89.1) |  |  | 93 (95.9) | 31 (88.6) |  |
| Grade > 2 |  | 1 (1.4) | 8 (12.5) |  |  | | 4 (4.4) | 5 (10.9) |  |  | 4 (4.1) | 4 (11.4) |  |
| Diarrhea |  |  |  | NS (.087) |  | |  |  | NS (>.999) |  |  |  | NS (.522) |
| Grade 0/1 |  | 69 (94.5) | 54 (84.4) |  |  | | 81 (90.0) | 41 (89.1) |  |  | 88 (90.7) | 30 (85.7) |  |
| Grade > 2 |  | 4 (5.5) | 10 (15.6) |  |  | | 9 (10.0) | 5 (10.9) |  |  | 9 (9.3) | 5 (14.3) |  |
| Fatigue |  |  |  | NS (.425) |  | |  |  | NS (>.999) |  |  |  | NS (.486) |
| Grade 0/1 |  | 58 (79.5) | 47 (73.4) |  |  | | 69 (76.7) | 36 (78.3) |  |  | 76 (78.4) | 25 (71.4) |  |
| Grade > 2 |  | 15 (20.5) | 17 (26.6) |  |  | | 21 (23.3) | 10 (21.7) |  |  | 21 (21.6) | 10 (28.6) |  |
| Mucositis/stomatitis |  |  |  | NS (.820) |  | |  |  | NS (.230) |  |  |  | NS (.433) |
| Grade 0/1 |  | 60 (82.2) | 54 (84.4) |  |  | | 72 (80.0) | 41 (89.1) |  |  | 79 (81.4) | 31 (88.6) |  |
| Grade > 2 |  | 13 (17.8) | 10 (15.6) |  |  | | 18 (20.0) | 5 (10.9) |  |  | 18 (18.6) | 4 (11.4) |  |
| Anorexia |  |  |  | NS (.166) |  | |  |  | NS (>.999) |  |  |  | NS (.650) |
| Grade 0/1 |  | 59 (80.8) | 45 (70.3) |  |  | | 68 (75.6) | 35 (76.1) |  |  | 74 (76.3) | 25 (71.4) |  |
| Grade > 2 |  | 14 (19.2) | 19 (29.7) |  |  | | 22 (24.4) | 11 (23.9) |  |  | 23 (23.7) | 10 (28.6) |  |
| **Treatment efficacy** |  | **Emotional (cut-off value = 83.3 )** | | |  | | **Cognitive (cut-off value = 83.3 )** | | |  | **Social (cut-off value = 66.7 )** | | |
|  |  | **High** | **Low** | ***P* value** |  | | **High** | **Low** | ***P* value** |  | **High** | **Low** | ***P* value** |
| Total No. (%) |  | 73 (53.3) | 64 (46.7) |  |  | | 98 (72.1) | 38 (27.9) |  |  | 120 (88.9) | 15 (11.1) |  |
| 2 year-OS (%) |  | 65.5 | 63.1 | NS (.788)* |  | | 65.2 | 62.2 | NS (.463)* |  | 63.6 | 78.8 | NS (.959)* |
| mPFS (months) |  | 10.3 | 10.5 | NS (.492)* |  | | 10.3 | 11.2 | NS (.686)* |  | 10.5 | 15.3 | NS (.931)* |
| 95% CI |  | 7.7-12.8 | 8.2-12.1 |  |  | | 8.0-12.0 | 8.2-13.1 |  |  | 9.5-11.8 | 1.6-20.6 |  |
| Best overall response: No. (%) |  |  |  | NS (>.999)** |  | |  |  | NS (.325)** |  |  |  | NS (>.999)** |
| CR/PR |  | 38 (52.0) | 35 (54.7) | NS (>.999)*** | | 49 (50.0) | | 23 (60.5) | NS (>.999)*** | | 64 (53.3) | 8 (53.3) | NS (.129)*** |
| SD |  | 24 (32.9) | 20 (31.3) |  |  | | 34 (34.7) | 10 (26.3) |  |  | 40 (33.3) | 3 (20.0) |  |
| PD |  | 6 (8.2) | 6 (9.4) |  |  | | 9 (9.2) | 3 (7.9) |  |  | 9 (7.5) | 3 (20.0) |  |
| NE |  | 5 (6.9) | 3 (4.7) |  |  | | 6 (6.2) | 2 (5.3) |  |  | 7 (5.8) | 1 (6.7) |  |
| Adverse events: No. (%) |  |  |  |  |  | |  |  |  |  |  |  |  |
| Skin reaction |  |  |  | NS (>.999) |  | |  |  | NS (.443) |  |  |  | NS (.786) |
| Grade 0/1 |  | 33 (45.8) | 29 (45.3) |  |  | | 47 (48.4) | 15 (39.5) |  |  | 55 (46.2) | 6 (40.0) |  |
| Grade > 2 |  | 39 (54.2) | 35 (54.7) |  |  | | 50 (51.6) | 23 (60.5) |  |  | 64 (53.8) | 9 (60.0) |  |
| Hepatic dysfunction |  |  |  | NS (>.999) |  | |  |  | NS (.944) |  |  |  | NS (.252) |
| Grade 0/1 |  | 62 (84.9) | 54 (84.4) |  |  | | 83 (84.7) | 32 (84.2) |  |  | 103 (85.8) | 11 (73.3) |  |
| Grade > 2 |  | 11 (15.1) | 10 (15.6) |  |  | | 15 (15.3) | 6 (15.8) |  |  | 17 (14.2) | 4 (26.7) |  |
| Renal dysfunction |  |  |  | NS (>.999) |  | |  |  | NS (.534) |  |  |  | NS (>.999) |
| Grade 0/1 |  | 66 (90.4) | 57 (89.1) |  |  | | 89 (90.8) | 33 (86.8) |  |  | 107 (89.2) | 14 (93.3) |  |
| Grade > 2 |  | 7 (9.6) | 7 (10.9) |  |  | | 9 (9.2) | 5 (13.2) |  |  | 13 (10.8) | 1 (6.7) |  |
| Electrolyte imbalance |  |  |  | NS (.840) |  | |  |  | NS (.362) |  |  |  | NS (.747) |
| Grade 0/1 |  | 55 (75.3) | 50 (78.1) |  |  | | 78 (79.6) | 27 (71.1) |  |  | 93 (77.5) | 11 (73.3) |  |
| Grade > 2 |  | 18 (24.7) | 14 (21.9) |  |  | | 20 (20.4) | 11 (28.9) |  |  | 27 (22.5) | 4 (26.7) |  |
| Neutropenia |  |  |  | NS (.609) |  | |  |  | NS (.056) |  |  |  | NS (.585) |
| Grade 0/1 |  | 35 (48.0) | 34 (53.1) |  |  | | 55 (56.1) | 14 (36.8) |  |  | 59 (49.2) | 9 (60.0) |  |
| Grade > 2 |  | 38 (52.0) | 30 (46.9) |  |  | | 43 (43.9) | 24 (63.2) |  |  | 61 (50.8) | 6 (40.0) |  |
| Anemia |  |  |  | NS (.478) |  | |  |  | **0.006** |  |  |  | NS (>.999) |
| Grade 0/1 |  | 29 (39.7) | 21 (32.8) |  |  | | 43 (43.9) | 7 (18.4) |  |  | 45 (37.5) | 5 (33.3) |  |
| Grade > 2 |  | 44 (60.3) | 43 (67.2) |  |  | | 55 (56.1) | 31 (81.6) |  |  | 75 (62.5) | 10 (66.7) |  |
| Nausea |  |  |  | NS (.334) |  | |  |  | NS (.793) |  |  |  | NS (.698) |
| Grade 0/1 |  | 60 (82.2) | 57 (89.1) |  |  | | 84 (85.7) | 32 (84.2) |  |  | 101 (84.2) | 14 (93.3) |  |
| Grade > 2 |  | 13 (17.8) | 7 (10.9) |  |  | | 14 (14.3) | 6 (15.8) |  |  | 19 (15.8) | 1 (6.7) |  |
| Vomiting |  |  |  | NS (.502) |  | |  |  | NS (.709) |  |  |  | NS (.597) |
| Grade 0/1 |  | 67 (91.8) | 61 (95.3) |  |  | | 92 (93.9) | 35 (92.1) |  |  | 111 (92.5) | 15 (100.0) |  |
| Grade > 2 |  | 6 (8.2) | 3 (4.7) |  |  | | 6 (6.1) | 3 (7.9) |  |  | 9 (7.5) | 0 (0.0) |  |
| Diarrhea |  |  |  | **0.009** |  | |  |  | NS (.349) |  |  |  | NS (.811) |
| Grade 0/1 |  | 61 (83.6) | 62 (96.9) |  |  | | 86 (87.8) | 36 (94.7) |  |  | 108 (90.0) | 13 (86.7) |  |
| Grade > 2 |  | 12 (16.4) | 2 (3.1) |  |  | | 12 (12.2) | 2 (5.3) |  |  | 12 (10.0) | 2 (13.3) |  |
| Fatigue |  |  |  | NS (>.999) |  | |  |  | NS (.656) |  |  |  | NS (>.999) |
| Grade 0/1 |  | 56 (76.7) | 49 (76.6) |  |  | | 76 (77.6) | 28 (73.7) |  |  | 91 (75.8) | 12 (80.0) |  |
| Grade > 2 |  | 17 (23.3) | 15 (23.4) |  |  | | 22 (22.4) | 10 (26.3) |  |  | 29 (24.2) | 3 (20.0) |  |
| Mucositis/stomatitis |  |  |  | NS (.255) |  | |  |  | NS (.613) |  |  |  | NS (>.999) |
| Grade 0/1 |  | 58 (79.5) | 56 (87.5) |  |  | | 80 (81.6) | 33 (86.8) |  |  | 99 (82.5) | 13 (86.7) |  |
| Grade > 2 |  | 15 (20.5) | 8 (15.5) |  |  | | 18 (18.4) | 5 (13.2) |  |  | 21 (17.5) | 2 (13.3) |  |
| Anorexia |  |  |  | NS (.690) |  | |  |  | NS (.505) |  |  |  | NS (.523) |
| Grade 0/1 |  | 54 (74.0) | 50 (78.1) |  |  | | 76 (77.6) | 27 (71.1) |  |  | 92 (76.7) | 10 (66.7) |  |
| Grade > 2 |  | 19 (26.0) | 14 (21.9) |  |  | | 22 (22.4) | 11 (28.9) |  |  | 28 (23.3) | 5 (33.3) |  |

**Abbreviations**: CR, complete response; PR, partial response; SD, stable disease; PD, progressive disease; NE, not evaluable; NS, not significant.

* log rank test was performed, ** CR/PR vs. SD/PD, *** CR/PR/SD vs. PD. Fisher's exact test was performed. A higher score represents better levels of GHS/QOL and functioning
